# To frame or not to frame? Cone‐beam CT‐based analysis of head immobilization devices specific to linac‐based stereotactic radiosurgery and radiotherapy

**DOI:** 10.1002/acm2.12251

**Published:** 2018-01-24

**Authors:** Steven Babic, Young Lee, Mark Ruschin, Fiona Lochray, Alex Lightstone, Eshetu Atenafu, Nic Phan, Todd Mainprize, May Tsao, Hany Soliman, Arjun Sahgal

**Affiliations:** ^1^ Department of Radiation Oncology Sunnybrook Odette Cancer Centre University of Toronto Toronto ON Canada; ^2^ Department of Biostatistics Princess Margaret Cancer Centre University of Toronto Toronto ON Canada; ^3^ Division of Neurosurgery Sunnybrook Health Sciences Centre University of Toronto Toronto ON Canada

**Keywords:** cranial stereotactic radiotherapy, frame‐based immobilization, precision frameless stereotaxy, radiosurgery

## Abstract

**Purpose:**

Noninvasive frameless systems are increasingly being utilized for head immobilization in stereotactic radiosurgery (SRS). Knowing the head positioning reproducibility of frameless systems and their respective ability to limit intrafractional head motion is important in order to safely perform SRS. The purpose of this study was to evaluate and compare the intrafractional head motion of an invasive frame and a series of frameless systems for single fraction SRS and fractionated/hypofractionated stereotactic radiotherapy (FSRT/HF‐SRT).

**Methods:**

The noninvasive PinPoint system was used on 15 HF‐SRT and 21 SRS patients. Intrafractional motion for these patients was compared to 15 SRS patients immobilized with Cosman‐Roberts‐Wells (CRW) frame, and a FSRT population that respectively included 23, 32, and 15 patients immobilized using Gill‐Thomas‐Cosman (GTC) frame, Uniframe, and Orfit. All HF‐SRT and FSRT patients were treated using intensity‐modulated radiation therapy on a linear accelerator equipped with cone‐beam CT (CBCT) and a robotic couch. SRS patients were treated using gantry‐mounted stereotactic cones. The CBCT image‐guidance protocol included initial setup, pretreatment and post‐treatment verification images. The residual error determined from the post‐treatment CBCT was used as a surrogate for intrafractional head motion during treatment.

**Results:**

The mean intrafractional motion over all fractions with PinPoint was 0.62 ± 0.33 mm and 0.45 ± 0.33 mm, respectively, for the HF‐SRT and SRS cohort of patients (*P*‐value = 0.266). For CRW, GTC, Orfit, and Uniframe, the mean intrafractional motions were 0.30 ± 0.21 mm, 0.54 ± 0.76 mm, 0.73 ± 0.49 mm, and 0.76 ± 0.51 mm, respectively. For CRW, PinPoint, GTC, Orfit, and Uniframe, intrafractional motion exceeded 1.5 mm in 0%, 0%, 5%, 6%, and 8% of all fractions treated, respectively.

**Conclusions:**

The noninvasive PinPoint system and the invasive CRW frame stringently limit cranial intrafractional motion, while the latter provides superior immobilization. Based on the results of this study, our clinical practice for malignant tumors has evolved to apply an invasive CRW frame only for metastases in eloquent locations to minimize normal tissue exposure.

## 1 INTRODUCTION

Accurate treatment positioning and patient immobilization is of the upmost importance for cranial stereotactic radiosurgery (SRS).^1^ Traditional approach is to use invasive head frame which can fix the cranium rigidly and no treatment margin beyond the treated target is needed.^2^, ^3^, ^4^ With noninvasive head immobilization devices, common practice has been to apply a small planning target volume (PTV) margin to account for uncertainties. Depending upon the immobilization device and image‐guidance system that is used to detect and correct for motion, PTV margins typically range from 1 to 3 mm. The clinical disadvantage of adding any PTV margin is an increased risk of radionecrosis as a result of a greater volume of normal tissue receiving the high dose.^5^

The drawbacks of using an invasive frame include patient anxiety, pain associated with placement of the screws which are typically applied to the outer table of the cranium, and risk of bleeding and infection at the site of placement.^6^ Compared to head frames, noninvasive immobilization systems such as thermoplastic masks have been shown to offer patient immobilization inferior to what is required for SRS, but sufficient for fully fractionated stereotactic radiotherapy (FSRT).^7^, ^8^, ^9^, ^10^, ^11^, ^12^ These immobilization systems have been increasingly used in hypofractionated stereotactic radiotherapy (HF‐SRT), stereotactic radiation delivered in 2 to 5 fractions, but the performance has not been well studied.^13^

Head immobilization technology has made significant advances by incorporating sophisticated mouth bite apparatus^14^, ^15^ and integrated vacuum suctioning system that (a) reduces air gaps between the hard palate and the mouth bite to reduce potential slippage and (b) warns the therapists if the patient head moves which causes the vacuum pressure to drop. While there exist a number of studies that have assessed the positional accuracy and stability of different invasive and noninvasive immobilization systems for cranial stereotactic radiotherapy,^6^, ^9^, ^16^, ^17^, ^18^, ^19^, ^20^ to our knowledge, no study has directly compared the performance of various systems used in the same institution.

The aim of the present study is to evaluate patient intrafraction head motion for a series of widely available invasive and noninvasive immobilization systems for SRS, HF‐SRT, and FSRT.

## 2 METHODS

### 2.A Cranial immobilization devices

**Figure 1:**
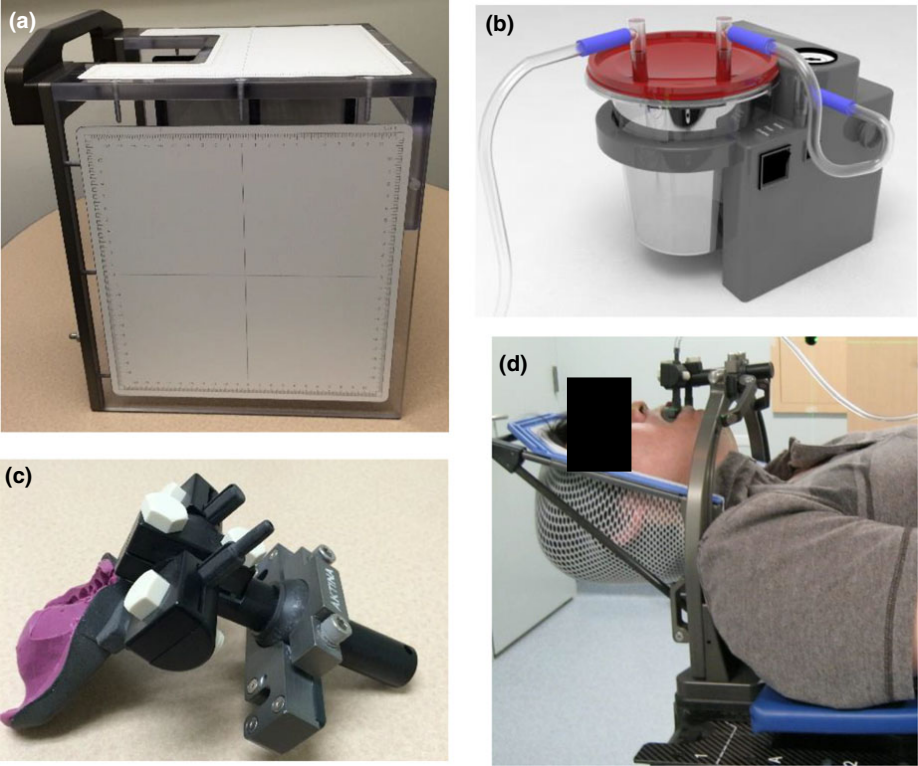
PinPoint system. (a) cranial patient setup box, (b) portable vacuum suction device, (c) vacuum fixation mouthpiece with patient‐specific dental impression and (d) thermoplastic support frame together with mouthpiece attached to an external arch block.

The head immobilizations under study included: the invasive Cosman‐Roberts‐Wells (CRW) frame (Integra‐Radionics, Burlington, MA, USA), relocatable Gill‐Thomas‐Cosman (GTC) frame (Integra‐Radionics, Burlington, MA, USA), thermoplastic Uniframe (WFR/Aquaplast Corp., Avondale, PA, USA), thermoplastic Orfit (Orfit Industries, Wijnegem, Belgium), and noninvasive PinPoint frame (Aktina Medical, Congers, NY, USA).^12^, ^18^, ^21^ Note that CRW was considered the “gold standard” in this study and the PinPoint system (shown in Fig. 1) was specifically acquired as an alternative to using the invasive CRW frame for SRS at our center. This device is equipped with a vacuum fixation bite‐block device consisting of an external and internal component that work in tandem such that patients cannot move their head without losing suction. The internal component contains a patient‐specific dental mouthpiece with continuous mild vacuum suction to the upper hard palate (assures tight contact). The external component consists of the dental mouthpiece secured to a metal arch frame that is in turn locked into a carbon‐fiber couch board equipped with a thermoplastic head support formed by creating an impression of the back of the skull (note that previous versions utilized an alpha‐cradle for head support^14^). A reference box with three embedded spherical radiopaque markers is attached over the bridge of the PinPoint system to assist patient setup.

### 2.B Treatment delivery

All HF‐FSRT and FSRT patients were treated using intensity‐modulated radiation therapy (IMRT) delivery with a 6 MV photon beam using an Elekta Synergy Beam Modulator (Elekta AB, Stockholm, Sweden) equipped with a 4 mm multileaf collimator, on‐board kilovoltage (kV) cone‐beam CT (CBCT), and a six degree‐of‐freedom (6‐DOF) robotic couch top (HexaPOD, Elekta AB, Stockholm, Sweden). The distance between the CBCT isocenter and the MV isocenter is measured through daily quality assurance tests and is stringently kept to within 1 mm. All SRS patients were treated on the same unit using stereotactic cones (Elekta AB, Stockholm, Sweden) externally mounted on the gantry head. For all treatment plans, coplanar and noncoplanar beam arrangements were used. The total treatment delivery time was on the order of 20 min for both HF‐FSRT and FSRT, and 20 min per isocenter for SRS (with each target having between 1 and 3 isocenters).

### 2.C Image‐guidance protocol

An initial CBCT scan was acquired after the patient was immobilized and positioned at the treatment isocenter using a localizer device (CRW), a setup reference box (PinPoint) or reference marks on the frame (GTC) and thermoplastic mask (Uniframe, Orfit). Registration to the planning CT, to determine the precise vector shift required to match the CBCT isocenter to the planning CT isocenter, was completed using grayscale matching. CBCT/CT image fusion accuracy has been shown to be <0.1 mm in each direction using the grayscale algorithm^21^ and a region of interest encompassing the PTV and cranium (Elekta X‐ray Volume Imaging (XVI) software v.4.0). Translational (X = lateral, Y = superior‐inferior, Z = anterior‐posterior) and rotational (pitch, roll, yaw) offsets were recorded and fine positioning corrections prior to treatment were made using the 6‐DOF HexaPOD robotic couch (Elekta AB, Stockholm, Sweden). Consistent with what others have achieved,^21^ HexaPOD was tested during commissioning and found to agree with software to within ±0.3 mm for all translations and ±0.2° for each rotational axis.

For this study, the repositioning threshold was strict at 1 mm and 1° in any translational or rotational axis, respectively since it has been shown that positioning the target to as close to the intended position as possible, that is, 1 mm threshold for patient repositioning, reduces subsequent out‐of‐tolerance motions and improves the overall precision in delivery.^22^ A pretreatment verification CBCT was taken to confirm that the isocenter was within a three‐dimensional (3D) vector magnitude of 1.5 mm of the pretreatment CT isocenter. At the end of treatment, the couch was returned to 0° and a post‐treatment CBCT acquired and registered with the treatment planning CT. All shifts were documented and a 3D vector positioning error was quantified using Eq. (1).

(1) $$\begin{array}{c} 3D\;positioning\;\;error=\\ \sqrt{Xdisplacement^{2}+Ydisplacement^{2}+Zdisplacement^{2}} \end{array}$$

For patients that were positioned within our 1 mm/1° tolerance based on pretreatment CBCT (and no pretreatment shifts applied), we used the difference between pre and post‐treatment shifts as the intrafractional motion. For the small subgroup of patients that were outside our 1 mm/1° tolerance and that required a second couch shift prior to treatment, the shifts generated from the post‐treatment CBCT were used as a surrogate of intrafractional motion. It is acknowledged that some portion of these shifts included the residual error of couch motion.

The 3D intrafraction displacement was calculated as the vector difference between pre and post‐treatment CBCTs. Note that time between pre and post‐CBCTs was 20 min and this was the same for both HF‐FSRT and FSRT (the total treatment time) and SRS (the time to treat a single isocenter).

### 2.D Patient population and treatment characteristics

The single fraction SRS patient population (treatment with 20 Gy in 1 fraction) included 15 patients immobilized with a CRW, and 21 patients immobilized using PinPoint. The total number of CBCT images for analysis was 45 and 63 for CRW and PinPoint, respectively.

The FSRT population included 16 five fraction HF‐SRT patients immobilized with PinPoint, and 23, 32, and 15 fully fractionated (1.8–2.0 Gy per day over 25 to 30 fractions) FSRT patients immobilized using GTC, Uniframe, and Orfit, respectively. The total number of treatment fractions delivered were 77, 403, 497, and 81 which resulted in 231, 1209, 1491, and 243 CBCT images for analysis based on immobilization with the PinPoint, GTC, Uniframe, and Orfit, respectively. Considering HF‐SRT, FSRT, and SRS, 3282 cone‐beam CT images in total were acquired and analyzed.

Although we pooled all of the data together, regardless of treatment technique (SRS/FSRT/HF‐SRT) and disease indication, the CRW frame was only used to immobilize brain metastases patients receiving SRS. GTC and thermoplastic masks were used to immobilize brain metastases or primary brain tumor patients receiving FSRT or HF‐SRT. The PinPoint system was originally evaluated in FSRT patients and subsequently used for single fraction SRS of brain metastases.

### 2.E Statistical analysis

For each immobilization device, the translational, rotational and calculated 3D positioning error from each treatment fraction was, respectively, grouped within the following stages of observation: initial setup, pretreatment, post‐treatment, and intrafractional motion. The mean and standard deviation were calculated from the entire set of all fractions tabulated within the respective translational, rotational, and 3D displacement error datasets.

Box plot was used to describe the distribution of positioning setup displacements for varying immobilization systems as well as for the 3D error. Line plot was also used to show the distribution of inverse cumulative frequency of intrafractional motion by device. Considering each treatment fraction as independent measurement, an analysis of variance (ANOVA) was used to compare on the different immobilization devices and their respective mean 3D error, translational, and rotational errors. If there was a significant difference between the immobilization devices, then a further pair‐wise comparison to the gold standard (CRW) was carried out after using the Benjamini & Hochberg^23^ correction for multiple comparisons. Results were considered significant if the adjusted *P*‐value was <0.05. An additional comparison between FSRT and SRS patients immobilized with the PinPoint was completed to determine any statistically significant differences (*P*‐value <0.05). Statistical analyses were performed using version 9.4 of the SAS system for Windows (2002–2012 SAS Institute, Inc., Cary, NC, USA).

## 3 RESULTS

### 3.A Initial positioning setup errors

**Figure 2:**
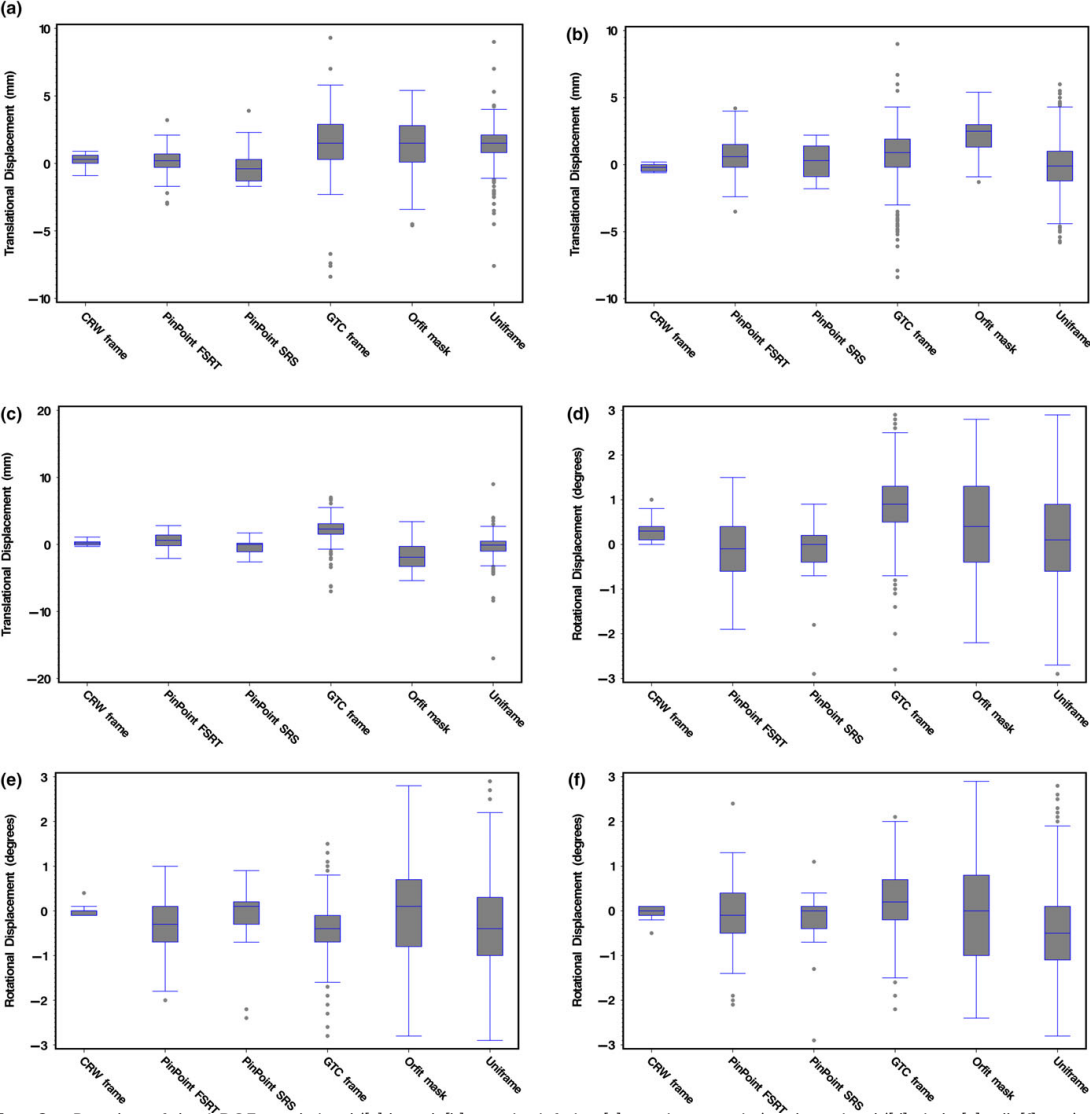
Box plots of the 6‐DOF translational ([a] lateral, [b] superior‐inferior, [c] anterior‐posterior) and rotational ([d] pitch, [e] roll, [f] yaw) initial positioning setup displacements for the immobilizations systems under study: CRW frame (*N* = 15), PinPoint SRS (*N* = 21), PinPoint HF‐SRT (*N* = 77), GTC frame (*N* = 403), Orfit mask (*N* = 81), and Uniframe (*N* = 497).

Initial positioning setup errors were analyzed to reveal the setup uncertainty of manual positioning of the different immobilization systems. The 6‐DOF translational and rotational displacements for each immobilization system are shown in Fig. 2 as standard box plots that display the full range of variation (from min to max), the likely range of variation (the interquartile range between first and third quartiles), and the typical value (median). Across all immobilization systems, the mean translational displacement in the lateral in Fig. 2(a), superior‐inferior in Fig. 2(b) and anterior‐posterior in Fig. 2(c) directions together with the mean rotational displacement in the pitch in Fig. 2(d), roll in Fig. 2(e) and yaw in Fig. 2(f) directions were found to be statistically significant but nonspecific.

**Figure 3:**
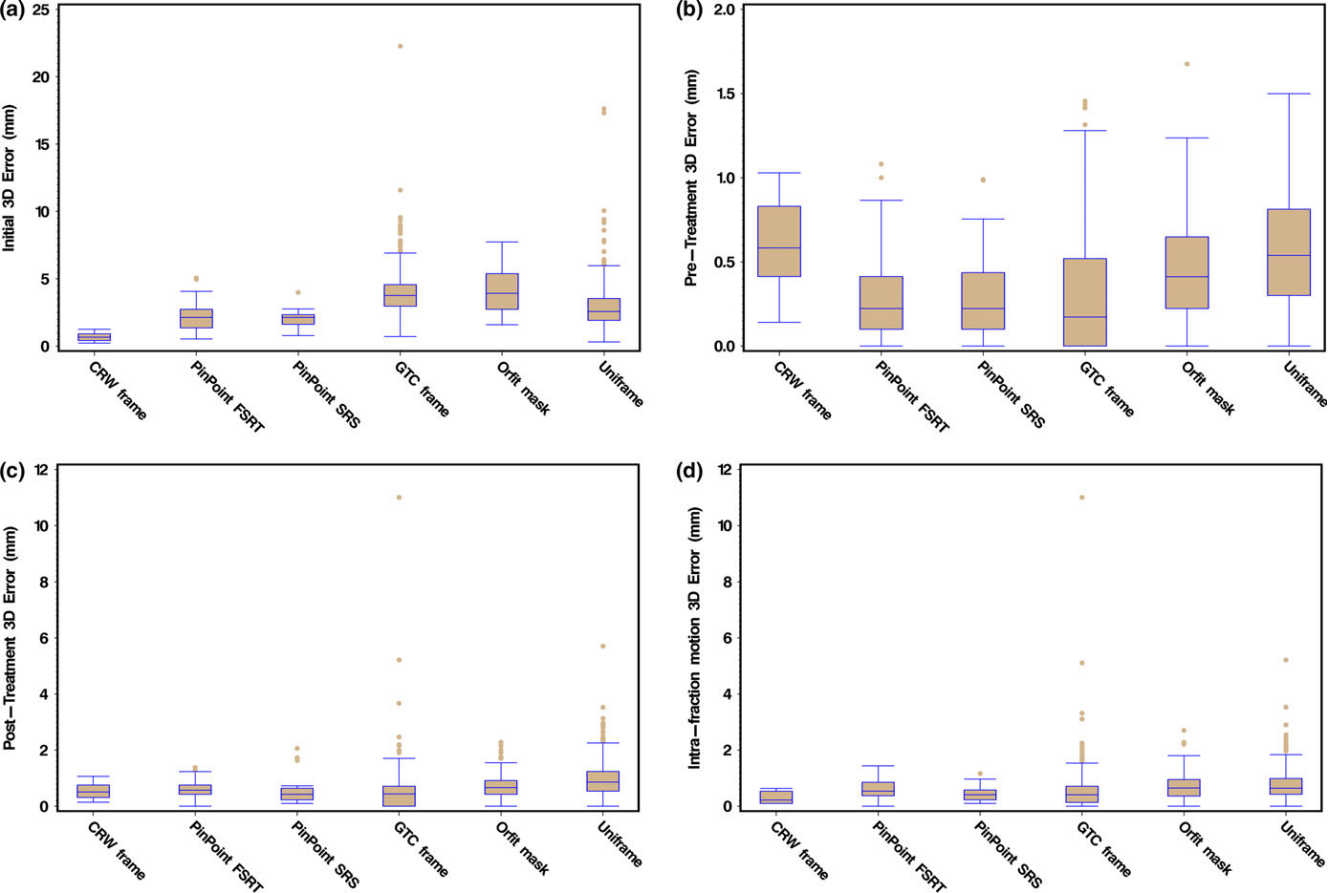
Presented as box plots, the 3D error (vector magnitude) at (a) setup, (b) just prior to treatment and (c) post‐treatment to adjust the anatomy for a given immobilization device to the planned treatment isocenter based upon CBCT/CT fusion. (d) For each immobilization system, the magnitude of 3D intrafraction movement is calculated from the vector difference between pre and the post‐treatment CBCTs.

The lowest initial 3D setup error was observed with the CRW frame (mean value of 0.67 mm) followed by the PinPoint system (mean values of 2.06 and 2.15 mm for SRS and HF‐SRT). The GTC frame and the frameless thermoplastic Uniframe and Orfit masks had the greatest mean 3D error and departure from zero (approximately double that of CRW and PinPoint) as summarized in Fig. 3(a). A further pair‐wise comparison to the gold standard CRW frame indicated that these results were statistically significant (adjusted *P*‐value <0.0025).

### 3.B Pretreatment residual errors

Due to the strict repositioning threshold set at 1 mm and 1° in any translational or rotational axis, respectively, all pretreatment 3D errors as shown in Fig. 3(b) were <1.5 mm (our cutoff for acceptability ensuring that anatomy was within this 3D vector distance of the CBCT isocenter).

### 3.C Post‐treatment residual errors and intrafractional motion

Post‐treatment mean translational residual errors were between −0.25 and 0.11 mm, and the mean rotational errors were between −0.20° and 0.33° for all devices. The differences were found to be significant in all 6‐DOF except in the lateral direction. Note that commissioning of the 6‐DOF robotic couch showed that the ability to reproduce was only 0.3 mm and 0.2° for all translational and rotational axes, respectively; hence, some of this could be folded into residual errors that were quantified using the post‐treatment CBCTs.

A further pair‐wise comparison to the gold standard CRW frame indicated statistical significance in the pitch direction for PinPoint HF‐SRT, GTC frame, Uniframe, and Orfit (adjusted *P*‐value <0.02). The post‐treatment 3D error, as shown in Fig. 3(c), revealed that the PinPoint and CRW frame had a similar and reduced variability compared to GTC frame, Orfit, and Uniframe.

**Figure 4:**
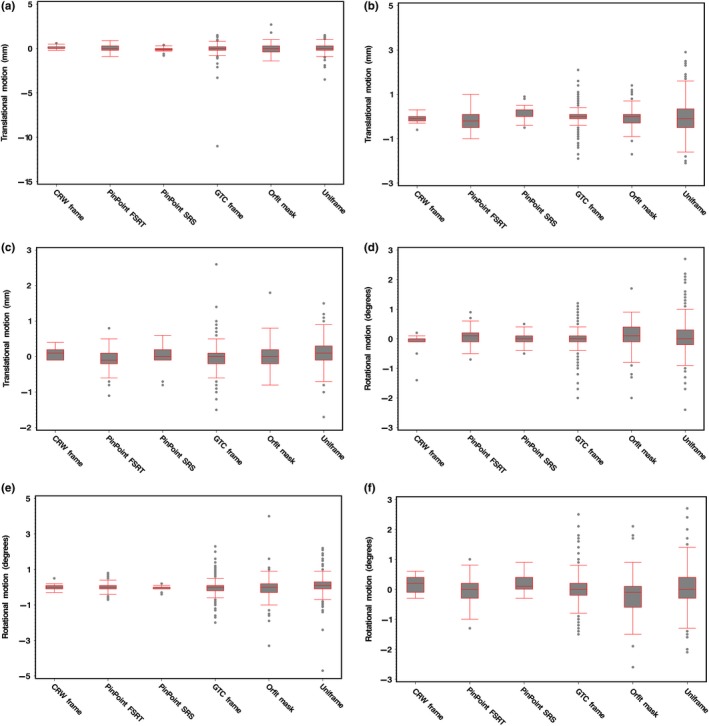
Box plots of the 6‐DOF translational ([a] lateral, [b] superior‐inferior, [c] anterior‐posterior) and rotational ([d] pitch, [e] roll, [f] yaw) intrafraction motion for the immobilizations systems under study: CRW frame (*N* = 15), PinPoint SRS (*N* = 21), PinPoint HF‐SRT (*N* = 77), GTC frame (*N* = 403), Orfit mask (*N* = 81), and Uniframe (*N* = 497).

Figure 4 summarizes the intrafractional motion data for all translations and rotations within each immobilization system, and Table 1 summarizes the corresponding mean displacement and 1 standard deviation. In the pitch direction in Fig. 4(d), the mean displacements were <0.12° and variability within ±1°. The largest amount of variability in both the roll in Fig. 4(e) and yaw in Fig. 4(f) directions was observed with the Orfit and Uniframe.

**Table 1:** Mean and standard deviations of intrafraction motion for all 6‐DOF translational and rotational displacements together with the 3D error for varying immobilization systems. Absolute maximum values and nonspecific statistical significance among all devices are highlighted in bold

Variable	3D Error (mm)	Lateral X (mm)	Sup‐Inf Y (mm)	Ant‐Post Z (mm)	Pitch (°)	Roll (°)	Yaw (°)
PinPoint HF‐SRT	0.62 (0.33)	0.01 (0.36)	−**0.17** (0.46)	−0.07 (0.33)	0.07 (0.27)	−0.02 (0.29)	−0.03 (0.43)
PinPoint SRS	0.45 (0.33)	−0.09 (0.28)	0.11 (0.35)	0.01 (0.32)	−0.00 (0.24)	−0.03 (0.13)	**0.22** (0.27)
GTC frame	0.54 (0.76)	−0.03 (0.74)	0.00 (0.43)	−0.04 (0.37)	−0.04 (0.35)	−0.01 (0.44)	0.02 (0.48)
Orfit mask	0.73 (0.49)	−0.04 (0.60)	−0.04 (0.51)	0.03 (0.39)	0.11 (0.54)	−0.06 (0.84)	‐0.17 (0.71)
Uniframe	**0.76** (0.51)	0.03 (0.43)	−0.04 (0.74)	**0.08** (0.34)	0.06 (0.51)	**0.08** (0.51)	0.06 (0.61)
CRW frame	0.30 (0.21)	**0.11** (0.22)	−0.09 (0.20)	0.07 (0.15)	−**0.12** (0.39)	0.03 (0.18)	0.12 (0.28)
*P*‐value	<**0.0001**	0.6224	0.2437	<**0.0001**	**0.0036**	<**0.0001**	**0.0049**

In the lateral direction in Fig. 4(a), there was no significant difference in the mean values, which for all devices were found to be near 0 mm except for the CRW frame which had a mean value of 0.11 mm, but showed the least amount of variability (PinPoint SRS exhibited the same). In the superior‐inferior direction in Fig. 4(b), PinPoint for both SRS and FSRT were both found to have the largest amount of mean residual error, −0.17 and 0.11 mm, respectively, but this was statistically nonsignificant. In the anterior‐posterior direction in Fig. 4(c), for all devices the mean values were near 0 mm (*P*‐value <0.0001).

With respect to mean intrafractional motion observed in the various systems (see Table 1), the CRW frame recorded the lowest value at 0.30 mm while the Uniframe and Orfit both had the largest at 0.76 and 0.73 mm, respectively. A pair‐wise comparison to the gold standard CRW frame indicated statistical significance in the mean intrafractional motion for only Uniframe (*P*‐value = 0.028) and Orfit (*P*‐value = 0.016). Although PinPoint HF‐SRT had a mean intrafractional motion of 0.62 mm as compared to PinPoint SRS with a value of 0.45 mm, the differences were not significant. As shown in Fig. 3(d), the CRW frame had the least variability in intrafractional motion compared to the other devices.

**Figure 5:**
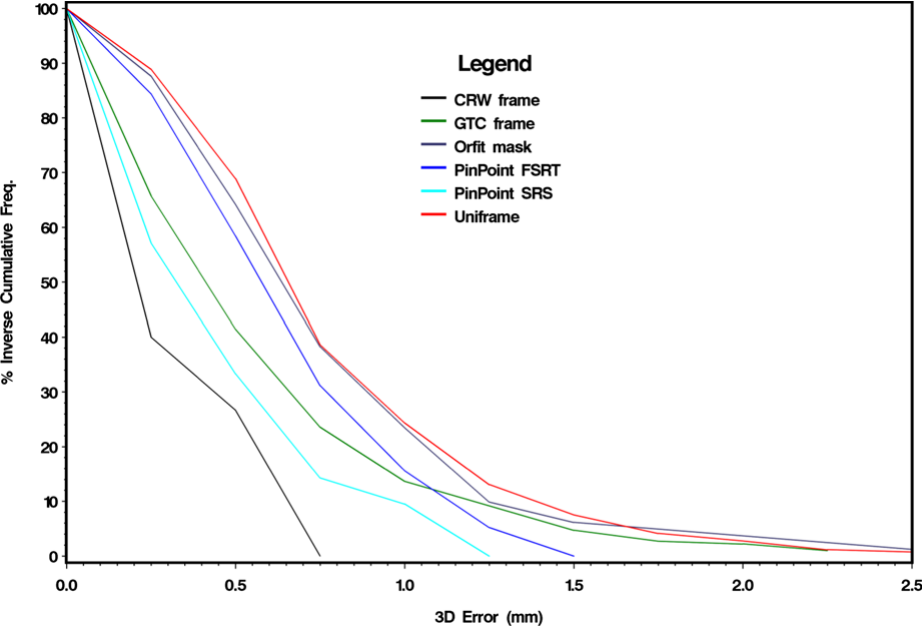
Distribution of inverse cumulative frequency of 3D error intrafraction motion by frame.

Figure 5 summarizes the distribution of percent inverse cumulative frequency of intrafractional motion, and it highlights that the least amount of motion was observed with CRW. With the CRW frame, intrafractional motion exceeded 0.5 mm in 27% of all fractions treated and there was no intrafractional motion beyond 0.75 mm. With PinPoint single fraction SRS versus HF‐SRT, intrafractional motion exceeded 1 mm in 10% and 16% of all fractions treated, respectively. There was no recordable intrafractional motion beyond 1.5 mm with PinPoint. For the GTC, Orfit, and Uniframe, intrafractional motion exceeded 1.5 mm in 5%, 6%, and 8% of all fractions treated, respectively. The percentage of fractions with intrafractional motion >2 mm was 2%, 3%, and 4% for GTC frame, Uniframe, and Orfit, respectively. For GTC frame and Orfit, there was no intrafractional motion beyond 2.5 mm. For Uniframe, 0.8% of all fractions treated exceeded 2.5 mm intrafractional motion, with no intrafractional motion beyond 2.75 mm.

## 4 DISCUSSION

Within this study, we report our institutional head immobilization experience with both frame‐based and frameless‐based SRS, HF‐SRT, and FSRT treatments on a linac equipped with a 6‐DOF robotic couch top and kV‐CBCT. Our motion analysis based on CBCT showed that PinPoint provides comparable immobilization compared to CRW, although CRW stills offer the least amount of intrafractional motion (<0.75 mm). When deciding to frame or not to frame for SRS, we have decided that a frame is required when a superior immobilization system is desired in cases where minimal exposure to the surrounding neural tissue is critical (e.g., lesions in the brainstem). Otherwise, we proceed with the PinPoint immobilization system for all single fraction SRS.

Despite being immobilized in an invasive stereotactic head frame, our analysis further showed that intrafractional cranial motion occurs with the CRW frame. This observation is consistent with results from Ramakrishna et al. who noted a mean intrafractional motion of 0.40 ± 0.30 mm with the invasive Brown‐Roberts‐Wells (BRW) head frame.^21^ Although the mean intrafractional motion of the CRW frame was only 0.30 ± 0.21 mm, and minimal when compared against the noninvasive immobilization systems evaluated in our study, it highlights the issue as to whether or not a PTV margin should be applied to invasive frame‐based SRS in order to ensure treatment efficacy. Note that the main purpose of this study was not to provide recommendations for PTV margins since intrafractional motion is only one component of many steps within the radiotherapy planning and delivery process that would go into determining the appropriate PTV margin.

In this study, we also observed that the mean intrafractional motion was found to be smaller in the SRS cohort of patients (0.45 ± 0.33 mm) versus HF‐SRT (0.62 ± 0.33 mm) patients immobilized with PinPoint. Although determined nonsignificant (*P*‐value = 0.266), these differences are particularly noticeable in the plot of the respective percent inverse cumulative frequencies between SRS versus HF‐SRT for intrafractional motion <1.0 mm (Fig. 5). Some of the differences may be attributable to HF‐SRT patients becoming more relaxed from the second fraction onwards. It is also possible that as the number of treatment fractions increases, the relative stability of the patient‐specific thermoplastic head support and mouthpiece in restricting head motion lessens. However, immobilization is still clinically acceptable since the amount of intrafractional motion exceeding 1.25 mm was 0% and 5% for SRS and HF‐SRT, respectively. With HF‐SRT patients, no intrafractional motion exceeded 1.5 mm (over a treatment delivery duration of 20 min). Note that in a separate analysis, we compared fraction 1 of the fractionated patients (HF‐SRT) to the results of the single fraction (SRS) patients and there was no statistically significant difference between the two that would indicate a bias in the patient setup or mask making.

The largest amount of intrafractional motion with the PinPoint system was observed in the superior‐inferior direction for both SRS and HF‐SRT patients. This was also observed by Li et al. who reported a slow head drifting motion in the longitudinal direction using a video‐based 3D optical surface imaging system.^14^ Li's study was limited by the evaluation of only two FSRT and two SRS patients (10 treatment fractions in total). Their mean 3D intrafraction translational and rotational motions were 0.3 ± 0.2 mm and 0.2° ± 0.1°, respectively. For 98% of the time, the magnitude of translational head motion with PinPoint was within 1.1 mm. Our results are based on a much more extensive analysis, with over 98 treatment fractions, and indicate a slightly greater magnitude of translational head motion at 1.4 mm. The differences may be due to the greater number of data points collected in our study as well the difference in the head support system (ours did not utilize an alpha‐cradle).

The GTC relocatable head frame had a mean intrafractional motion comparable to that of PinPoint. This is not unexpected since both of them are similar in design such that they are both based on a bite‐block device. However, with PinPoint's bite‐block, a gentle vacuum suction is applied between the dental mouthpiece and the upper hard palate to assure tight contact, and if the seal is broken a loud hissing sound is heard to alert both the patient and radiation therapist. This added mechanism to ensure stability likely explains the significant reduction in translational and rotational variability during initial setup (Fig. 2) and intrafractional motion (Fig. 4). From Fig. 5, at a cumulative frequency of ≤1.0 mm, the intrafractional motion for GTC lies between both PinPoint HF‐SRT and SRS. With PinPoint, there was no intrafractional motion >1.5 mm. With GTC frame, there was intrafractional motion >1.5 mm in ~5% of all fractions treated. Note that an intrafractional motion >10 mm was also observed with the GTC frame. The outlier is from a single patient (treated on their 6^th^ fraction out of 25) whose post‐treatment CBCT analysis indicated a 10 mm shift in the lateral (X) direction. This particular patient was treated in February 2010 and access to the CBCT images to verify this unexpected large lateral shift is no longer available. Since we cannot determine if the outlying point is “real” or due to a transcription error, the data point was not excluded.

The largest initial setup error and intrafractional motion was observed with both thermoplastic mask systems, Uniframe and Orfit. Although the setup error was large, CBCT image‐guidance together with the HexaPOD significantly improved the positioning accuracy. While the measured intrafractional motion (mean 3D vector = 0.76 ± 0.51 mm and 0.73 ± 0.49 mm for Uniframe and Orfit, respectively) is close to being adequate for frameless‐based SRS (for the CRW frame the mean intrafractional motion = 0.30 ± 0.21 mm), the percentage of fractions with intrafractional motion >2 mm was 3% and 4% for Uniframe and Orfit, respectively, which is suboptimal for SRS. Similar to our study, Masi et al. utilized kV‐CBCT image‐guidance to quantify the initial setup error and intrafractional motion of a simple thermoplastic mask (Novastereo; Novater, Milano, Italy) used to immobilize 17 patients for 35 fractions.^12^ The mean 3D setup error was 3.2 ± 1.5 mm and the mean intrafractional motion was ~0.4 mm.

Since the residual error determined from the post‐treatment CBCT was used as an indication (or surrogate) of patient motion during treatment (referred to as intrafractional motion), one limitation of this study is that we are only interrogating a single time point and we do not have a complete description of how well the patient remained immobilized during the actual treatment. With CBCT, one cannot directly track patient movement during treatment and it is only capable of verifying patient immobilization at 0° couch angle, i.e., possible patient motion at noncoplanar couch positions cannot be evaluated.

Not all known commercially available cranial immobilization devices were analyzed in this study. Gevaert et al. investigated the setup errors and intrafractional motion of 40 patients immobilized with the BrainLAB frameless mask system.^24^ Prior to 6‐DOF correction, the mean 3D setup error was 1.91 ± 1.25 mm, and the mean 3D intrafractional motion was determined to be 0.58 ± 0.42 mm. Using post‐treatment kV imaging, Ramakrishna et al. also investigated the intrafractional motion with the BrainLAB mask and found a mean intrafraction shift of 0.7 ± 0.5 mm.^6^ Ruschin et al. reported on the setup accuracy and intrafractional motion of the eXtend frame system on 12 patients treated on a linear accelerator and Gamma Knife machine (Perfexion, Elekta, Stockholm, Sweden).^15^ Similar in design to PinPoint, eXtend is a noninvasive vacuum bite‐block repositioning head frame. Utilizing CBCT, the mean intrafractional motion was found to be 0.4 ± 0.3 mm. In order to ensure the high accuracy and precision required for SRS particularly when frameless‐based localization and fixation is used, both our results and those from literature^15^, ^25^ indicate that 3D image‐guidance is essential.

## 5 CONCLUSIONS

PinPoint and CRW frame deliver stringent immobilization. Although intrafractional cranial motion is also observed with the invasive CRW frame, it yields the least amount of intrafractional motion and provides superior head immobilization compared to PinPoint and mask‐based immobilization systems. The latter noninvasive systems always require image‐guidance verification in order to ensure the high accuracy and precision needed for SRS. When deciding to frame or not to frame, our results influenced our practice such that unless lesions are in eloquent tissues where minimal exposure to the surrounding neural tissue is critical, the head immobilization system of choice for SRS is the noninvasive PinPoint.

## CONFLICT OF INTEREST

Dr. Arjun Sahgal has received an honorarium for previous education seminars and research funding from Elekta AB. Drs. Lee, Ruschin and Soliman are participants on a research group sponsored by Elekta AB.

## References

[1] Schell MC , Bova FJ , Larson DA , et al. Stereotactic radiosurgery In American Association of Physicists in Medicine Report No. 54. Woodbury, NY: American Institute of Physics, 1995:1–88.

[2] Lightstone AW , Benedict SH , Bova FJ , Solberg TD , Stern RL . Intracranial stereotactic positioning systems: Report of the American Association of Physicists in Medicine Radiation Therapy Committee Task Group No. 68. Med Phys. 2005;32:2380–2398. 10.1118/1.1945347 16121596

[3] Lundsford LD , Leksell D . The Leksell system In Modern Stereotactic Neurosurgery. Boston, MA: Martinus Nijhoff Publishing 1988:−.

[4] Lutz W , Winston KR , Maleki N . A system for stereotactic radiosurgery with a linear accelerator. Int J Radiat Oncol Biol Phys. 1988;14:373–381. 3276655 10.1016/0360-3016(88)90446-4

[5] Kirkpatrick JP , Wang Z , Sampson JH , et al. Defining the optimal planning target volume in image‐guided stereotactic radiosurgery of brain metastases: results of a randomized trial. Int J Radiat Oncol Biol Phys. 2015;91:100–108. 25442342 10.1016/j.ijrobp.2014.09.004

[6] Ramakrishna N , Rosca F , Friesen S , Tezcanli E , Zygmanszki P , Hacker F . A clinical comparison of patient setup and intra‐fraction motion using frame‐based radiosurgery versus a frameless image‐guided radiosurgery system for intracranial lesions. Radiother Oncol. 2010;95:109–115. 20116123 10.1016/j.radonc.2009.12.030

[7] Pan H , Cervino LI , Pawlicki T , et al. Frameless, real‐time, surface imaging‐guided radiosurgery: clinical outcomes for brain metastases. Neurosurg. 2012;71:844–851. 10.1227/NEU.0b013e3182647ad5 22989959

[8] Feygelman V , Walker L , Chinnaiyan P , Forster K . Simulation of intrafraction motion and overall geometric accuracy of a frameless intracranial radiosurgery process. J Appl Clin Med Phys. 2008;9:68–86. 10.1120/jacmp.v9i4.2828 PMC5722363 19020489

[9] Lightstone AW , Tsao M , Basran PS , et al. Cone beam CT (CBCT) evaluation of inter‐ and intra‐fraction motion for patients undergoing brain radiotherapy immobilized using a commercial thermoplastic mask on a robotic couch. Technol Cancer Res Treat. 2012;11:203–209. 22376132 10.7785/tcrt.2012.500288

[10] Fuss M , Salter BJ , Cheek D , Sadeghi A , Hevezi JM , Herman T . Repositioning accuracy of a commercially available thermoplastic mask system. Radiother Oncol. 2004;71:339–345. 15172151 10.1016/j.radonc.2004.03.003

[11] Guckenberger M , Roesch J , Baier K , Sweeney RA , Flentje M . Dosimetric consequences of translational and rotational errors in frame‐less image‐guided radiosurgery. Radiother Oncol. 2012;7:63. 10.1186/1748-717X-7-63 PMC3441228 22531060

[12] Masi L , Casamassima F , Polli C , Menichelli C , Bonucci I , Cavedon C . Cone beam CT image guidance for intracranial stereotactic treatments: comparison with a frame guided set‐up. Int J Radiat Oncol Biol Phys. 2008;71:926–933. 18514784 10.1016/j.ijrobp.2008.03.006

[13] Al‐Omair A , Soliman H , Xu W , et al. Hypofractionated stereotactic radiotherapy in five daily fractions for post‐operative surgical cavities in brain metastases patients with and without prior whole brain radiation. Technol Cancer Res Treat. 2013;12:493–499. 23617283 10.7785/tcrt.2012.500336 PMC4527429

[14] Li G , Ballangrud A , Kuo LC , et al. Motion monitoring for cranial frameless stereotactic radiosurgery using video‐based three‐dimensional optical surface imaging. Med Phys. 2011;38:3981–3994. 21858995 10.1118/1.3596526

[15] Ruschin M , Nayebi N , Carlsson P , et al. Performance of a novel repositioning head frame for gamma knife perfexion and image‐guided linac‐based intracranial stereotactic radiotherapy. Int J Radiat Oncol Biol Phys. 2010;78:306–313. 20385456 10.1016/j.ijrobp.2009.11.001

[16] Bednarz G , Machtay M , Werner‐Wasik M , et al. Report on a randomized trial comparing two forms of immobilization of the head for fractionated stereotactic radiotherapy. Med Phys. 2009;36:12–17. 19235368 10.1118/1.3030950

[17] Gill SS , Thomas DG , Warrington AP , Brada M . Relocatable frame for stereotactic external beam radiotherapy. Int J Radiat Oncol Biol Phys. 1991;20:599–603. 1995547 10.1016/0360-3016(91)90076-g

[18] Graham JD , Warrington AP , Gill SS , Brada M . A non‐invasive, relocatable stereotactic frame for fractionated radiotherapy and multiple imaging. Radiother Oncol. 1991;21:60–62. 1852920 10.1016/0167-8140(91)90342-e

[19] Ohtakara K , Hayashi S , Tanaka H , et al. Clinical comparison of positional accuracy and stability between dedicated versus conventional masks for immobilization in cranial stereotactic radiotherapy using 6‐degree‐of‐freedom image guidance system‐integrated platform. Radiother Oncol. 2012;102:198–205. 22100656 10.1016/j.radonc.2011.10.012

[20] Theelen A , Martens J , Bosmans G , et al. Relocatable fixation systems in intracranial stereotactic radiotherapy. Accuracy of serial CT scans and patient acceptance in a randomized design. Strahlenther Onkol. 2012;188:84–90. 22194025 10.1007/s00066-011-0018-7

[21] Meyer J , Wilbert J , Baier K , et al. Positioning accuracy of cone‐beam computed tomography in combination with a HexaPOD robot treatment table. Int J Radiat Oncol Biol Phys. 2007;67:1220–1228. 17336222 10.1016/j.ijrobp.2006.11.010

[22] Hyde D , Lochray F , Korol R , et al. Spine stereotactic body radiotherapy utilizing cone‐beam CT image‐guidance with a robotic couch: intrafraction motion analysis accounting for all six degrees of freedom. Int J Radiat Oncol Biol Phys. 2011;82:e555–e562. 10.1016/j.ijrobp.2011.06.1980 22284042

[23] Benjamini Y , Hochberg Y . Controlling the false discovery rate: a practical and powerful approach to multiple testing. J R Statist Soc B. 1995;57:289–300.

[24] Gevaert T , Verellen D , Engels B , et al. Clinical evaluation of a robotic 6‐degree of freedom treatment couch for frameless radiosurgery. Int J Radiat Oncol Biol Phys. 2012;83:467–474. 21945110 10.1016/j.ijrobp.2011.05.048

[25] Ali I , Tubbs J , Hibbitts K , et al. Evaluation of the setup accuracy of stereotactic radiotherapy head immobilization mask system using kV on‐board imaging. J Appl Clin Med Phys. 2010;11:26–37. 10.1120/jacmp.v11i3.3192 PMC5720447 20717086

